# The foliar application of a mixture of semisynthetic chitosan derivatives induces tolerance to water deficit in maize, improving the antioxidant system and increasing photosynthesis and grain yield

**DOI:** 10.1038/s41598-019-44649-7

**Published:** 2019-06-03

**Authors:** Valquíria Mikaela Rabêlo, Paulo César Magalhães, Letícia Aparecida Bressanin, Diogo Teixeira Carvalho, Caroline Oliveira dos Reis, Decio Karam, Antônio Carlos Doriguetto, Marcelo Henrique dos Santos, Plínio Rodrigues dos Santos Santos Filho, Thiago Corrêa de Souza

**Affiliations:** 1 Federal University of Alfenas – UNIFAL-MG, Institute of Natural Sciences- ICN,700, Gabriel Monteiro Street, P. O. Box 37130-001 Alfenas, MG Brazil; 2 Maize and Sorghum National Research Center, P. O. Box 151, 35701-970 Sete Lagoas, MG Brazil; 3 Federal University of Alfenas - UNIFAL-MG, Faculty of Pharmaceutical Sciences, 700, Gabriel Monteiro da Silva Street, P.O. Box 37130-001 Alfenas, MG Brazil; 4 Federal University of Alfenas - UNIFAL-MG, Chemistry Institute, Gabriel Monteiro da Silva Street, 700, P.O. Box 37130-001 Alfenas, MG Brazil; 5 Federal University of Viçosa – UFV, Chemistry Departament, Peter Henry Rolfs Street, s/n, P.O. Box 36570-000 Viçosa, MG Brazil; 6 Federal University of Alfenas - UNIFAL-MG, Biochemistry Departament, Gabriel Monteiro da Silva Street, 700, P.O. Box 37130-001 Alfenas, MG Brazil; 7 Federal University of Alfenas – UNIFAL-MG, Institute of Natural Sciences- ICN, 700, Gabriel Monteiro Street, P. O. Box 37130-001 Alfenas, MG Brazil

**Keywords:** Plant physiology, Drought

## Abstract

Research has shown that chitosan induces plant stress tolerance and protection, but few studies have explored chemical modifications of chitosan and their effects on plants under water stress. Chitosan and its derivatives were applied (isolated or in mixture) to maize hybrids sensitive to water deficit under greenhouse conditions through foliar spraying at the pre-flowering stage. After the application, water deficit was induced for 15 days. Analyses of leaves and biochemical gas exchange in the ear leaf were performed on the first and fifteenth days of the stress period. Production attributes were also analysed at the end of the experiment. In general, the application of the two chitosan derivatives or their mixture potentiated the activities of the antioxidant enzymes superoxide dismutase, catalase, ascorbate peroxidase, glutathione reductase and guaiacol peroxidase at the beginning of the stress period, in addition to reducing lipid peroxidation (malonaldehyde content) and increasing gas exchange and proline contents at the end of the stress period. The derivatives also increased the content of phenolic compounds and the activity of enzymes involved in their production (phenylalanine ammonia lyase and tyrosine ammonia lyase). Dehydroascorbate reductase and compounds such as total soluble sugars, total amino acids, starch, grain yield and harvest index increased for both the derivatives and chitosan. However, the mixture of derivatives was the treatment that led to the higher increase in grain yield and harvest index compared to the other treatments. The application of semisynthetic molecules derived from chitosan yielded greater leaf gas exchange and a higher incidence of the biochemical conditions that relieve plant stress.

## Introduction

The increase in the world population, associated with the increase in agricultural production, has caused a series of negative environmental changes to the planet. Climatic changes lead to severe droughts that interfere with the physiological cycle of many cultivated plants. In addition, high temperatures have reached most maize-producing countries. Maize is a crop of great economic importance, as it is used for both human and animal consumption. Abiotic stresses such as drought are considered the most limiting crop production factors around the world^1^.

Water stress is among the most severe abiotic stresses for maize production. A few days of water deficit in the vegetative stage leads to a decrease in photosynthetically active leaf area in addition to inhibition of root growth. At the flowering stage, this abiotic stress can decrease grain yield and can reach losses of 20 to 50% due to floral abortion, silk desiccation, poor floral synchronization and a decrease in the formation of sinks^2^.

Most regions that grow maize do not have irrigation technologies available. In the world in general, as well as in Brazil, irrigation is not a totally viable technique, since it is necessary to seek sustainable alternatives that minimize water use in agricultural crops. This type of crop is always exposed and dependent on water-related factors^3^.

Soil water limitation induces plant stress, decreases cell volume, inhibits Calvin cycle enzymes, causes damage to the photosynthetic apparatus, decreases carbon assimilation and, consequently, decreases yield^4^.

Radiation in plants with inhibited of Calvin cycle enzymes (due to drought) causes excess energy in the antenna complex of photosystems, a process by which chlorophylls receive a large amount of energy and photoprotective pigments such as carotenoids and xanthophylls, preventing them from dissipating photochemical energy in the form of heat, thus dissipating this energy to oxygen, forming reactive oxygen species (ROS), which, in large amounts, generate oxidative stress that can lead to plant death^5^.

ROS are found at small concentrations in cellular organelles, such as chloroplasts, mitochondria or peroxisomes, or as by-products of photosynthesis, photorespiration or respiration^5^. However, in water stress and in the presence of radiation, the production of these compounds is exaggerated, leading to damage to the plasma membrane (lipid peroxidation) and degradation of pigments, proteins and DNA^6^.

The control of stable ROS levels in cell compartments and in stress situations is or may be maintained by the action of enzymatic (superoxide dismutase, ascorbate peroxidase, catalase, glutathione reductase, polyphenol oxidase, L-phenylalanine ammonia lyase, guaiacol peroxidase and dehydroascorbate reductase) and non-enzymatic (phenolic compounds, α-tocopherol, ascorbic acid, among others) antioxidant mechanisms^7,8^.

To control water stress, plants also activate osmotic adjustment mechanisms, an efficient physiological process for cellular turgescence, under conditions of low soil water potential^9^. This mechanism leads to the accumulation of solutes such as proline, glycine betaine, trehalose, and sucrose, among others, in the cytosol or cell vacuole^10^.

In search of sustainable agriculture techniques with less impact on the environment, research has sought to relieve water stress with the use of biopolymers such as chitin/chitosan, as they have low or no toxicity to the environment and can yield tolerance to water deficits^11^. Chitin is the second most abundant polysaccharide in nature, and its deacetylation results in chitosan, which has been used on several agricultural crops, including maize^12,13^, due to its potential as an elicitor, and its antifungal, protective and water stress relief abilities^14,15^.

Chitosan has a nucleophilic behaviour, making it suitable for structural chemical modifications; among these main modifications, acetylation, quaternization, alkylation, carboxylation, acylation, sulfonation and amidation can be obtained^16^. The synthesis of chitosan derivatives by the insertion of functional groups into the polymer chain gives this polymer different properties, allowing its use in medical, biotechnological, and agricultural areas^14,15,17^. However, in agriculture, few studies are based on chitosan derivatives. These derivatives are molecules that can potentiate activity since chitosan itself has important functions in the induction of tolerance to water deficit^18^.

Therefore, considering the economic importance of the maize crop and the constant harvest losses due to the water resource, the objective of this study was to apply new chitosan derivatives (N-succinyl and N, O-dicarboxymethylated) and to evaluate gas exchange, the antioxidant system and the primary metabolism in maize hybrids sensitive to water deficit.

In this study, we hypothesized that the application of N-succinyl chitosan and N, O- dicarboxymethylated chitosan derivatives or the mixture of the two would be capable of inducing tolerance to water deficit in stress-sensitive maize, associated with effective antioxidant protection through enzymatic and non-enzymatic defence mechanisms.

## Results

### Quantification of malonaldehyde (MDA) and the enzymatic system

**Figure 1:**
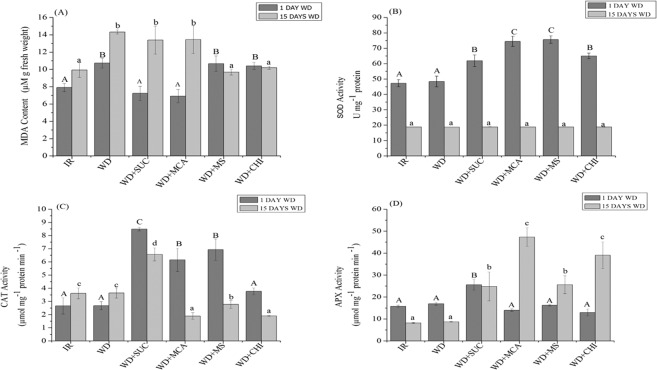
Concentration of MDA (**A**) and activity of the enzymes superoxide dismutase (SOD) (**B**), catalase (CAT) (**C**) and ascorbate peroxidase (APX) (**D**) during 15 days of water deficit in the maize hybrid BRS 1030 after the application of chitosan and its derivatives. Means followed by the same uppercase letter for treatments with 1 day of water deficit and lowercase for treatments with 15 days of water deficit do not differ by the Scott-Knott test at 5% probability (P ≤ 0.05). Each bar indicates the mean ± S.E. IR, irrigated; WD, water deficit; WD + SUC, water deficit with foliar application of SUC; WD + MCA, water deficit with foliar application of MCA; WD + MS, water deficit with foliar application of SUC and MCA; WD + CHI, water deficit with foliar application of chitosan.

On the first day of water deficit imposition, the content of MDA was reduced in the treatments with the application of the derivatives (WD + SUC) and (WD + MCA) when compared to the WD treatment (Fig. 1A). After 15 days of water deficit, the content of MDA was reduced in the treatments with the application of the mixture of the derivatives (WD + MS) and chitosan (WD + CHI).

For the antioxidant system, it can be observed that the activity of SOD (Fig. 1B) increased with the application of the MCA derivative and the mixture of the two derivatives, followed by chitosan and the SUC derivative. After 15 days of water deficit imposition, the activity of the enzyme did not show significant differences among treatments. On the other hand, the activity of the enzyme catalase (CAT) (Fig. 1C) with one day of water deficit increased in treatments (WD + SUC), (WD + MCA) and (WD + MS), and after 15 days, catalase had its highest activity in the treatment (WD + SUC) when compared with WD.

At the beginning of the water deficit, the enzyme APX (Fig. 1D) showed a higher activity in treatment (WD + SUC). At the end of the water deficit (15 days), APX showed an increase in its activity in all treatments with the application of the derivatives and chitosan.

**Figure 2:**
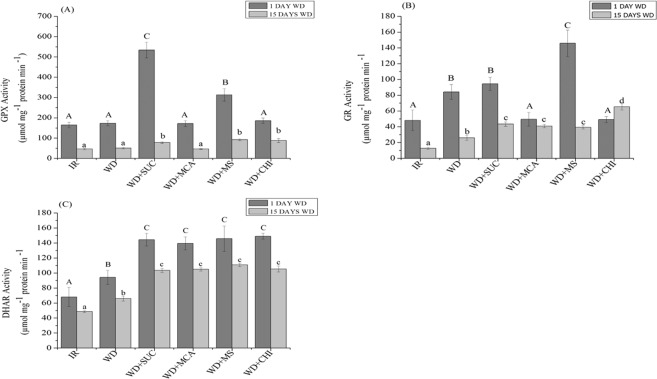
Activity of guaiacol peroxidase (GPX) (**A**), glutathione reductase (GR) (**B**) and dehydroascorbate reductase (DHAR) (**C**) during 15 days of water deficit in the maize hybrid BRS 1030 after the application of chitosan and its derivatives. Means followed by the same uppercase letter for treatments with 1 day of water deficit and lowercase for treatments with 15 days of water deficit do not differ by the Scott-Knott test at 5% probability (P ≤ 0.05). Each bar indicates the mean ± S.E. IR, irrigated; WD, water deficit; WD + SUC; water deficit with foliar application of SUC, WD + MCA, water deficit with foliar application of MCA; WD + MS, water deficit with foliar application of SUC and MCA; WD + CHI, water deficit with foliar application of chitosan.

The activity of GPX (Fig. 2A) increased with the application of the derivatives (WD + SUC) and (WD + MS) after 1 day of water deficit when compared to treatment (WD). With 15 days of water deficit, GPX activity increased in the (WD + SUC), (WD + MS) and (WD + CHI) treatments. The activity of GR (Fig. 2B) after 1 day of water deficit increased only in the mixture of the derivatives (WD + MS); however, after 15 days, GR activity increased in all treatments with the application of the derivatives and chitosan. The activity of DHAR (Fig. 2C) increased in all treatments with the application of chitosan and its derivatives on the first and last days of water deficit.

**Figure 3:**
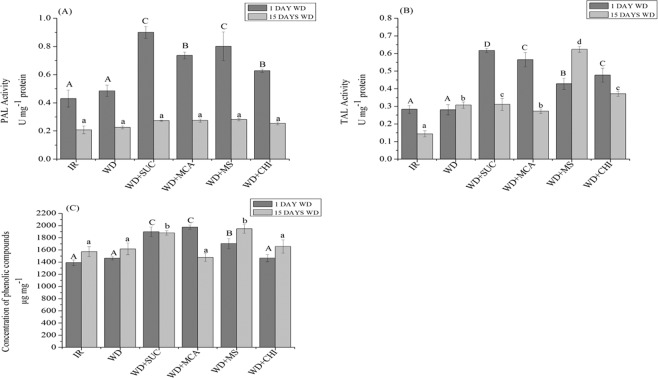
Activity of L-phenylalanine ammonia lyase (PAL) (**A**), tyrosine ammonia lyase (TAL) (**B**) and concentration of phenolic compounds (**C**) during 15 days of water deficit in the maize hybrid BRS 1030 after the application of chitosan and its derivatives. Means followed by the same uppercase letter for treatments with 1 day of water deficit and lowercase for treatments with 15 days of water deficit do not differ by the Scott-Knott test at 5% probability (P ≤ 0.05). Each bar indicates the mean ± S.E. IR, irrigated; WD, water deficit; WD + SUC, water deficit with foliar application of SUC; WD + MCA, water deficit with foliar application of MCA; WD + MS, water deficit with foliar application of SUC and MCA; WD + CHI, water deficit with foliar application of chitosan.

With the imposition of 1 day of water deficit, the enzymes PAL (Fig. 3A) and TAL (Fig. 3B) showed increased activity in all the treatments with the application of the derivatives and of chitosan and showed higher means for the plants that received SUC (WD + SUC) and the mixture (WD + SUC + MCA). On the 15th day of water deficit imposition, the activity of PAL did not show a significant difference among treatments, whereas TAL enzyme showed more activity in the treatments (WD + SUC), followed by (WD + MS) and (WD + CHI).

Phenolic compounds (Fig. 3C) had a higher concentration in the treatments (WD + SUC), (WD + MCA) and (WD + MS) after 1 and 15 days of water deficit when compared to the treatment (DW).

### Gas exchange analysis

After 1 day of water deficit, the application of the derivatives and chitosan did not increase the photosynthetic rate (A) (Table 1) in the hybrid BRS 1030 when compared to the irrigated treatment (IR). On the 15th day of water deficit imposition, it was observed that the photosynthetic rate decreased in the treatment (DW). However, the treatments (WD + SUC), (WD + MCA) and (WD + MS) increased the photosynthetic rate compared to the treatment (WD). For stomatal conductance (gs) (Table 1), the same effect was observed as that for the photosynthetic rate, with a decrease in the treatments with derivatives and chitosan at 1 day of water deficit. On the 15th day of water deficit, there was an increase in stomatal conductance for the treatments (WD + SUC), (WD + MCA) and (WD + MS) compared to treatment (WD).

**Table 1:** Leaf gas exchange during 15 days of water deficit in the maize hybrid BRS 1030 after the application of chitosan and its derivatives.

Treatment Stress Days	A (μmol CO_2_ m^−2^s^−1^) 1 day WD	gs (mol m^−2^ s^−1^) 1 day WD	A (μmol CO_2_ m^−2^s^−1^) 15 days WD	gs (mol m^−2^ s^−1^) 15 days WD
IR	28.7E*	0.29E	27.77c	0.32c
WD	26.39E	0.16C	14.23a	0.19b
WD + SUC	21.2D	0.24D	19.89b	0.46e
WD + MCA	2.64ª	0.04A	22.77b	0.41d
WD + MS	7.02B	0.07B	22.16b	0.23b
WD + CHI	10.81C	0.06B	16.1a	0.15a

*Means followed by the same uppercase letter for treatments with 1 day of water deficit and lowercase for treatments with 15 days of water deficit do not differ by the Scott-Knott test at 5% probability (P ≤ 0.05). IR, irrigated; WD, water deficit; WD + SUC, water deficit with foliar application of SUC; WD + MCA, water deficit with foliar application of MCA; WD + MS, water deficit with foliar application of SUC and MCA; WD + CHI, water deficit with foliar application of chitosan. A = leaf photosynthetic rate, gs = stomatal conductance.

### Quantification of proline and hydrogen peroxide (H_2_O_2_)

**Figure 4:**
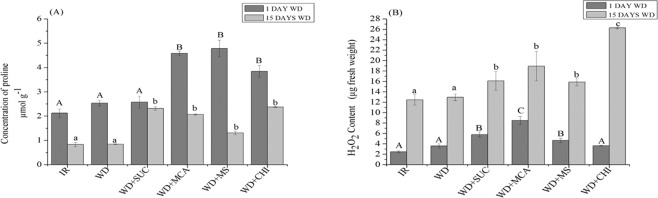
Concentration of proline (**A**) and content of hydrogen peroxide (H_2_O_2_) (**B**) during 15 days of water deficit in the maize hybrid BRS 1030 after the application of chitosan and its derivatives. Means followed by the same uppercase letter for treatments with 1 day of water deficit and lowercase for treatments with 15 days of water deficit do not differ by the Scott-Knott test at 5% probability (P ≤ 0.05). Each bar indicates the mean ± S.E. IR, irrigated; WD, water deficit; WD + SUC, water deficit with foliar application of SUC; WD + MCA, water deficit with foliar application of MCA; WD + MS, water deficit with foliar application of SUC and MCA; WD + CHI, water deficit with foliar application of chitosan.

The proline content (Fig. 4A) increased in treatments (WD + MCA), (WD + MS) and (WD + CHI) with the imposition of 1 day of water deficit. On the 15th day, all treatments with the application of the derivatives and chitosan showed a higher concentration of this osmoregulator when compared to treatment (WD).

The concentrations of H_2_O_2_ increased in treatments (WD + SUC), (WD + MCA) and (WD + MS) after 1 day of water deficit. At the end of the water deficit, all treatments with the application of the derivatives and chitosan showed higher H_2_O_2_ concentrations when compared to WD (Fig. 4B).

### Quantification of total soluble sugars, total amino acids and starch

**Figure 5:**
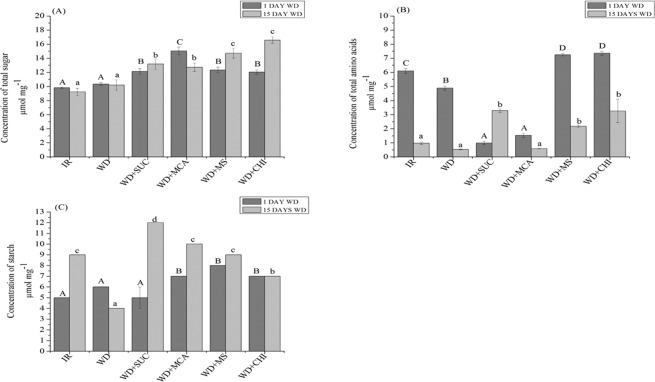
Concentration of total soluble sugars (**A**), total amino acids (**B**) and starch (**C**) during 15 days of water deficit in the maize hybrid BRS 1030 after the application of chitosan and its derivatives. Means followed by the same uppercase letter for treatments with 1 day of water deficit and lowercase for treatments with 15 days of water deficit do not differ by the Scott-Knott test at 5% probability (P ≤ 0.05). Each bar indicates the mean ± S.E. IR, irrigated; WD, water deficit; WD + SUC, water deficit with foliar application of SUC; WD + MCA, water deficit with foliar application of MCA; WD + MS, water deficit with foliar application of SUC and MCA; WD + CHI, water deficit with foliar application of chitosan.

When analysing the total soluble sugars (Fig. 5A), a higher concentration was observed in all treatments with the application of the derivatives and chitosan at 1 and 15 days of imposition of water deficit. The total amino acids (Fig. 5B) showed a higher concentration in the treatments (WD + SUC), (WD + MS) and (WD + CHI) with 1 day of water deficit. On the 15th day of water deficit, the highest concentrations were found in the treatments (WD + SUC), (WD + MS) and (WD + CHI). The starch content (Fig. 5C) was evidenced at higher concentrations in treatments (WD + MCA), (WD + MS) and (WD + CHI) with 1 day of water deficit. On the 15th day of water stress imposition, all treatments with the application of the derivatives and chitosan had a higher concentration of starch when compared to treatment (WD).

### Production components

It was observed that ear weight was higher in the irrigated treatment, and there was no difference between treatments stressed and stressed with the application of biopolymers (Table 2). Irrigated plants presented higher grain yield. However, the plants treated with the mixture of the derivatives (WD + MS) presented higher grain yield than those sprayed with chitosan or the separately applied derivatives. The same result observed in this parameter was also found in the harvest index (Table 2).

**Table 2:** Averages of maize production components (BRS 1030) under water deficit after application of chitosan and its derivatives.

Treatment Stress Days	Ear weight (g)	Grain yield (g plant^−1^)	Harvest index
IR	60.74 b*	39.92 d	0.49 d
WD	38.91 a	26.01 a	0.29 a
WD + SUC	44.34 a	28.23 b	0.34 b
WD + MCA	56.02 a	29.11 b	0.38 b
WD + MS	57.38 a	35.09 c	0.43 c
WD + CHI	49.14 a	28.89 b	0.35 b

*Means followed by the same lowercase letter for treatments do not differ by the Scott-Knott test at 5% probability (P ≤ 0.05). IR, irrigated; WD, water deficit; WD + SUC, water deficit with foliar application of SUC; WD + MCA, water deficit with foliar application of MCA; WD + MS, water deficit with foliar application of SUC and MCA; WD + CHI, water deficit with foliar application of chitosan.

## Discussion

Water stress is among the most severe stresses on maize production. A few days of water stress at the flowering stage decrease maize yield^18^. Water deficit causes several reactions in the plant, decreasing its water status (water potential), damaging and altering its photosystems, and increasing reactive oxygen species (ROS), causing degradation of lipid membranes^19^.

This degradation of the lipid membrane (lipid peroxidation) can be observed by the formation of a secondary metabolite known as MDA. Sensitive maize genotypes under water deficit have a higher MDA content^20–22^. The application of the N-succinyl and N, O-dicarboxymethylated derivatives decreased lipid peroxidation, showing more induction of water deficit tolerance than its initial structure, chitosan. The application of pure chitosan has relieved lipid peroxidation in maize, potato and peach plants^23–25^.

In maize plants tolerant to water deficit, one of the physiological responses to this exposure is the activation of the enzymatic antioxidant system to scavenge ROS^20,26^. However, sensitive maize genotypes tend not to exhibit this ability to scavenge ROS, in addition to presenting low photosynthetic rates when exposed to extended stress^19,20^. However, studies have shown that the application of chitosan can induce this antioxidant defences in plants in addition to increasing carbon assimilation^27–30^.

In this study, higher antioxidant enzymatic activity (SOD, CAT, APX, GPX and GR) was observed when the chitosan derivatives or their mixture were applied. This increase in enzymatic activity could explain the lower cell damage (lipid peroxidation), higher photosynthetic rate and stomatal conductance in these treatments. In a certain way, chitosan derivatives may have induced a greater tolerance to the hybrid BRS 1030 due to its performance in the physiological and molecular leaf processes.

Studies have shown that chitosan may act on nucleus and chloroplast genes involving increased photosynthetic and enzymatic antioxidant activities^31,32^. Chitosan has positive ionic charges, which gives it the ability to bind easily with negatively charged lipids, metal ions, proteins, and macromolecules. Although it has positive ionic charges, chitosan activates defence genes through changes in chromatin^33^. Chitosan has nucleotide sugars (uridine diphosphate N-acetyl-d-glucosamine (UDP-GlcNAc)) in its chemical constitution. When these sugars are applied in plants, the plant cell recognizes this site through the enzymes chitin synthase and chitosan chitin deacetylase. These enzymes recognize the β-1,4-linked N-acetylglucosamine residues and cleave these regions, producing chitosan oligomers that are important signals for plant cells^33,34^.

These chitosan oligomers arrive in the nucleus and in the chloroplastida and act in cascade reactions, inducing oxidative bursts and the production of hormones and modifying the chromatin and expression of antioxidant enzymes and photosynthetic enzymes^28^^,^^31,33,35–37^. A possible reason for the better response of the derivatives analysed in this study is that the oligosaccharides from the cleavage of the MCA and SUC derivatives containing N-succinyl and N, O-dicarboxymethylated groups by chitinases appear to be more active in potentiating enzymatic responses in sensitive maize than oligomers originating only from chitosan. In addition, the new chemical groups that were added to chitosan for the synthesis of derivatives tend to render these compounds more soluble in water^38^ and may also favour bioavailability.

The increase in H_2_O_2_ at the beginning of the water deficit in maize plants may be related to the role of chitosan (and its derivatives) in stress signalling and stomatal limitation (decrease in gs under drought)^38,39^. The initial production of H_2_O_2_ in maize may also be correlated with the activation of antioxidant enzymes^20^. Chitosan is considered a promoter of stomatal closure in the initial stages of water stress^40,41^, a fact observed in this study at higher proportions for plants treated with SUC and MCA derivatives on the first day of water deficit. This stomatal limitation occurs once chitosan can induce the production of stress signals such as H_2_O_2_ and abscisic acid (ABA)^41–44^.

In this study, an association of increased activity of phenylalanine ammonia lyase (PAL) and tyrosine ammonia lyase (TAL) was observed with the increase in the concentration of phenolic compounds. In addition, it is possible to highlight the higher TAL activity at the end of the water stress in the treatment in which the derivatives were applied and higher grain yield occurred. PAL and TAL are enzymes located between the primary and secondary metabolites and are related to the synthesis of the phenylpropanoid pathway, which is important for the synthesis of phenolic compounds^45^. These enzymes are considered the most important in the regulation of secondary metabolism. PAL is responsible for the production of trans-cinnamic acid and TAL for p-coumaric acid. These acids are incorporated into the formation of different phenolic compounds, which are present in the formation of esters, coumarins, flavonoids and lignins^45,46^. Maize-tolerant genotypes tend to present higher amounts of these compounds and especially TAL may be involved in this higher production^47,48^.

Several studies have shown that chitosan promoted increased activity of TAL, PAL and phenolic compounds in the plant antioxidant defence system against abiotic and biotic stresses^27,49–51^.

In this study, SUC and MCA derivatives, besides their mixture induced the production of phenolic compounds, and this provided an important increase in the tolerance of water deficit, even for maize^47,52^. Phenolic compounds such as flavonoids act in free radical scavenging and protection against oxidative stress in the face of water deficit^20,47,52^.

Both proline and sugars are important osmoregulators in maize, allowing water to remain inside foliar cells, even under water deficit conditions^53–55^. Chitosan and its derivatives induced greater accumulation of these compounds, favouring the tolerance of the sensitive hybrid (BRS 1030) to water deficit. Under water deficit conditions, the exogenous application of chitosan triggered in *Trifolium repens* a cascade of reactions that led to a greater tolerance of water deficit. In this experiment, chitosan yielded a greater accumulation of amino acids, sugars, starch, and flavonoids, among other metabolites. These compounds are associated with osmotic adjustment and the antioxidant defences of plants under stress conditions^56^. Plants that received the chitosan derivatives had higher amounts of amino acids in the leaves, which implies greater nitrogen uptake and/or mobilization or greater efficiency in the use of this nutrient in the plants that may have been favoured by the exogenous reception of the chitosan derivatives.

It is noteworthy that even at the end of 15 days of water deficit (lower water status), the plants that received the chitosan derivatives had greater leaf gas exchange (higher A and gs) and proline and sugars may facilitate this physiological stress response.

With respect to the effect of stimulating plant growth and development, in addition to modulating the expression of photosynthetic genes and redox homeostasis, chitosan can modulate carbohydrate metabolism^31,57^. For increased survival and growth of plants under stress, it is important to obtain energy and products from primary metabolism. Chitosan may increase the expression of the genes of enzymes involved in glycolysis^31^. The foliar application of the derivatives and chitosan yielded greater production of soluble sugars and starch, which are also inducers of carbon metabolism for tolerance to water stress. This higher amount of soluble sugars and starch could have been facilitated by the application of these biopolymers, as there was also greater photosynthesis with the application of these compounds.

Although the sprayed derivatives alone had an elicitor effect on the antioxidant system and on carbon metabolism, they did not result in production higher than that seen in the chitosan treatment. The treatment with biostimulants that was more relevant to the performance of the maize plant under water stress was the mixture of derivatives, as it resulted in a higher yield of grains. One of the reasons that may have led to a better yield from the mixture of the derivatives is the increase in the harvest index, which means a greater differential allocation of photoassimilates to the ear during the maize life cycle. Drought may lead to source and sink limitations, i.e., to decreased photosynthesis and/or ability to transport photoassimilates to the grains^2,19^. This difference in photoassimilate allocation and the photosynthetic rate between treatments shows that chitosan derivatives together may alleviate both source limitations and sink limitations, resulting in increased production.

The response of the plants to the mixture of derivatives suggests a synergistic effect when the two derivatives are applied together. The SUC and MCA derivatives have carboxylic acid groups that facilitate the solvation process in water and are therefore much more soluble in water than chitosan^58^. By leaving the two derivatives together in a mixture, they can form more ionized groups in the form of carboxylates, which would enhance their solubilization in the cellular medium and could explain their effects.

The higher effects of chitosan derivatives on oxidative stress responses were recorded mainly after application, i.e., at the beginning of the stress period but were also detected at the end of the stress period (15 days of stress). However, at the end of the stress period, although the spray treatments stand out in relation to the treatment without pulverization, the results are similar to those from pure chitosan. Thus, chitosan appears to act mainly at the end of the stress period, and the derivatives appear to act at the beginning and end of the stress period. This ensures protection for the plants during the whole stress period and can help explain the higher yield, especially in the mixture treatments. The mixture of derivatives resulted in a greater number of stress responses, which may be related to the increase in the enzymes SOD, CAT, GPX, GR, and PAL, and the increase in the content of phenolic compounds and proline. The greater stress response could also help explain the higher yield in the mixture treatments.

It is worth noting that the mixture of derivatives resulted in a significant/strong increase of glutathione reductase (GR) at the beginning of water stress. The mixture of derivatives increased the activity of an enzyme that has been shown to be essential in tolerance to abiotic stress, including drought^59^. GR is responsible for the regeneration of glutathione (GSH) using an electron donated by NADPH^60^. The GR in corn plants is present in three isoforms: one in the cytosol and two in the mitochondria and chloroplast^61^. As the chitosan oligomers can act as a signal in the chloroplastídeos^31,33,36^ where the highest GR activity is found (about 80% of the GR activity in photosynthetic tissues occurs by the chloroplast isoform)^61,62^, the oligomers of derived from chitosan that are formed in the form of a mixture could be potentializing the activity of this enzyme and contributing to the increase of grain yield. In addition, H_2_O_2_, which also increased in our work with the application of the mixture of derivatives at the beginning of stress, has been shown to be an ABA inducer that activates GR^62^. Several studies have shown the important role of GR in tolerance to water deficit in maize by reducing oxidative damages^63–65^.

The faster the plant stress signalling and the activation of the antioxidant defence begins, the less damage will be caused by a drought. Research on maize and sugarcane varieties with contrasting tolerances with spraying of salicylic acid also found an increase in antioxidant defence in the initial and final stages of the water deficit^66,67^. This does not occur with all biostimulants, which shows the particularity and importance of the studied derivatives. In sensitive maize, for example, abscisic acid did not lead to biochemical changes at the onset of stress^20^.

## Conclusion

Under greenhouse conditions, the BRS 1030 hybrid under water deficit showed induced tolerance to the effects of water restriction and increased production attributes due to foliar application of a mixture of chitosan derivatives (N-succinyl and N, O- dicarboxymethylated). Such an induction of tolerance is due to the increase in photosynthesis and the activity of the antioxidant enzymes that minimize the oxidative effects caused by ROS. Chitosan and derivatives (applied separately) also induced the antioxidant defence system and increased the attributes of production but were inferior to the mixture of derivatives.

The higher activity of enzymes PAL and TAL also yielded a higher concentration of phenolic compounds in maize. The derivative application also resulted in greater foliar gas exchange and higher concentrations of osmoregulators, such as sugars and proline, which are important in preserving water status. All of these associated factors show that these molecules (N-succinyl and N, O-dicarboxymethyl) can minimize the cell damage caused by water deficit and improve the physiological and biochemical conditions of a stress-sensitive hybrid, making it more tolerant.

## Methods

### Synthesis of chitosan derivatives

The N-succinyl derivative was prepared according to Li and Ding^68^, with modifications. Chitosan (1 g) (Galena Química e Farmacêutica Ltda) was dissolved with magnetic stirring at room temperature in 100 mL of 1% (v/v) glacial acetic acid solution. Subsequently, a solution of succinic anhydride (1.8 g) in acetone (20 mL) was added dropwise under stirring. The mixture formed was subjected to ultrasonic irradiation of 40–50 Hz in a bath at 50 °C for 60 minutes. The resulting solution was then cooled to room temperature, hydrated ethyl alcohol (100 mL) was added, and the mixture was transferred to a freezer, where it remained for 24 hours. After this period, 1 mol L^−1^ aqueous sodium hydroxide solution was added until pH = 10. Subsequently, acetone was added until precipitation occurred as a whitish caseous mass. The mixture was again conditioned in a freezer for 48 hours. After this period, the product was filtered under vacuum using ethyl alcohol (approximately 1000 mL) to wash the retained solid, which was stirred with a glass stick throughout the cleaning process. The final product was obtained as an amorphous, coarse and yellowish-white solid after drying in a desiccator under vacuum and being protected from light. Using the same procedure, additional quantities of the product were obtained.

The N, O-dicarboxymethylated derivative was prepared according to the methodology proposed by Liu *et al*.^69^. Chitosan (5 g) was added to isopropyl alcohol (60 mL) under magnetic stirring at room temperature. An aqueous sodium hydroxide solution (12 mL) was then added at 10 mol L^−1^, divided into five portions, over a period of 25 minutes. The mixture was magnetically stirred for 30 minutes at room temperature. Subsequently, monochloroacetic acid (30 g) was added, divided into five portions over five minutes. The formed mixture was heated at 70 °C under magnetic stirring for 3 hours. The reaction mixture was then cooled, and the obtained solid product was vacuum-filtered and washed with absolute methanol (100 mL).

**Figure 6:**
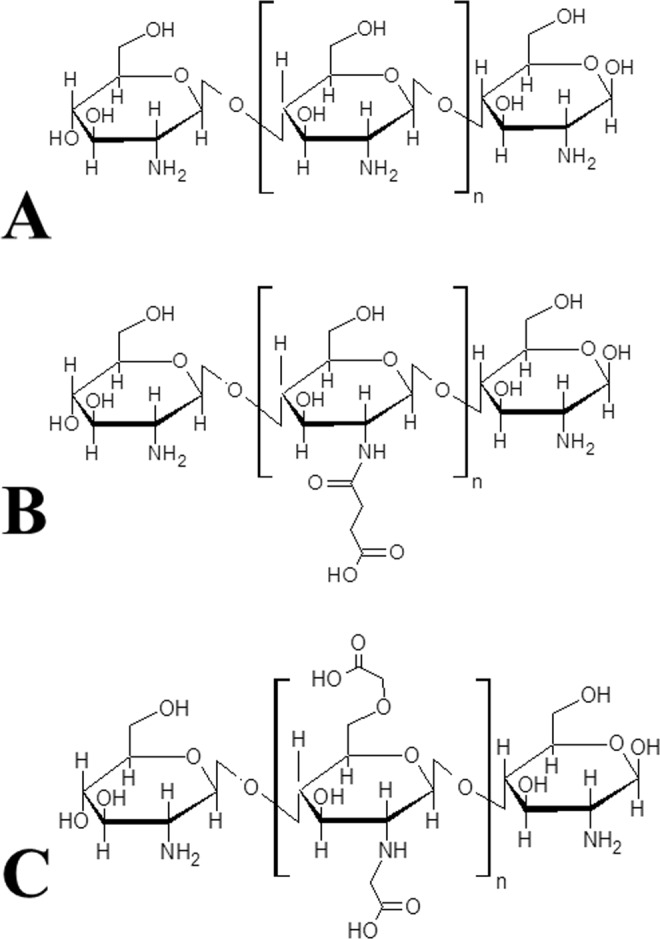
Chemical structure of chitosan (CHI) (**A**); of the N-succinyl derivative (SUC) (**B**); and of the N, O-dicarboxymethylated derivative (MCA) (**C**).

The product was rapidly oven-dried at 60 °C, which resulted in a yellow solid. Additional quantities of the product were obtained using the same procedure. The chitosan used to obtain the derivatives has a percentage of deacetylation (DDA%) of 63.5%^17^. The structures of the derivatives and chitosan are shown in Fig. 6.

### Plant material and growth conditions

A hybrid sensitive to water deficit (BRS 1030)^18^ from the Embrapa Breeding Programme was used. The experiment was conducted in a greenhouse located at Embrapa National Maize and Sorghum Research Centre in the city of Sete Lagoas, MG (732 m altitude, latitude 19°28′S, longitude 44°15′W). The average temperatures recorded during the evaluation period were as follows: maximum 36.3 °C, minimum 17.6 °C. Relative air humidity ranged from 72% to 40%.

The experiment was conducted in 20-L pots pre-filled with Typical Dystrophic Red Latosol. Fertilization was performed according to the soil chemical analysis recommendation, applying 10 g of 08-28-16 per 20 kg of soil at planting. Nitrogen side dressing was applied using 6 g of ammonium sulfate per pot at 30 and 60 days after planting. Three seeds were planted per pot, and after germination, thinning was performed, leaving two plants per pot. The plants were regularly irrigated, maintaining good soil moisture until water stress imposition.

### Water stress imposition, application of chitosan and derivatives

The soil water potential was monitored daily in the morning and afternoon (9.00 a.m. and 3.00 p.m.), with the aid of a tensiometer (Watermark 200SS, Irrometer, California, USA), installed in the centre of the pots of each replicate at a depth of 20 cm. Water was replaced based on the readings obtained with the sensor and then returned to field capacity (FC) during the period that preceded the treatments. These calculations were performed with the aid of a spreadsheet made according to the water retention curve of the soil.

At the pre-flowering stage, two water treatments were imposed: irrigated (IR) and water deficit (WD). The first treatment consisted of daily irrigation until the soil reached a moisture close to field capacity (FC) (soil water tension of approximately −0.18 MPa), whereas in WD, irrigation was performed applying 50% of the total water available, that is, until the water tension in the soil reached −1.38 MPa, whose value corresponds to the soil specified.

The preparation of the solutions was made from the dilution of 0.5 mg of chitosan and its derivatives in 250 mL of water at a ratio of 0.5 mg/plant. Chitosan and its derivatives (SUC and MCA) and their mixture at the same ratio (MS) were applied using a costal sprayer with a flow rate of 120 Lha^−1^. Each plant was sprayed at a concentration of 0.5 mg plant^−1^ through a pressurized CO_2_ costal sprayer (2.15 kg f cm^−2^) equipped with an XR – Teejet 110.02 VS nozzle, spraying the equivalent of 120 L ha^−1^. This spraying occurred one day before water deficit imposition (55th day after planting) at the pre-flowering stage, and the application was made from outside the greenhouse to avoid receiving the product by other treatments. This procedure was standardized and timed as shown in the video (Supplementary Material, Video 1) simulating field application.

### Gas exchange measurements

Gas exchange was measured on the 1st and 15th days of water stress imposition through a portable photosynthesis system (IRGA, LI-6400 XT, Li-Cor, Lincoln, Nebraska, USA). All measurements were taken in the morning, between 8.00 and 10.00 a.m. on a fully expanded leaf (spike leaf). The variables evaluated were photosynthetic rate (A) and stomatal conductance (g_s_). Measurements were taken in a leaf area of 6 cm^2^, with controlled CO_2_ flux at a concentration of 380 µmol CO_2_ mol^−1^ air. The photon flux density (PPFD) was 1500 µmol m^−2^ s^−1^ with a blue-red LED light source (6400-02B LED) and a controlled leaf temperature (30 °C)

### Material collection for biochemical analysis

For biochemical analyses, the spike leaf was collected on the first and last day of water deficit imposition, submerged in liquid nitrogen and frozen at −80 °C.

### Extraction and quantification of malonaldehyde (MDA) and hydrogen peroxide (H_2_O_2_)

Five hundred milligrams of plant material were macerated in liquid nitrogen; 2.5 mL of 0.1% trichloroacetic acid was added, and the material was centrifuged at 4,000 rpm for 30 minutes at 4 °C. Hydrogen peroxide was quantified according to the methodology proposed by Alixieva *et al*.^70^, and MDA (lipid peroxidation) was quantified according to Cakma and Horst^71^.

### Extraction and quantification of antioxidant enzymes

For enzyme extraction, 500 mg of plant material macerated in liquid nitrogen was used. Extraction was carried out with 50 mM potassium phosphate buffer, pH 7.5. In the extraction solution, 1 mM EDTA, 1 mM PMSF and 5% PVPP were added. The samples were centrifuged for 10 minutes at 14,000 rpm at 4 °C, and the supernatants were collected for quantification. The activity of the enzymes was expressed in milligrams (mg) of protein, which was determined by the Bradford method^72^.

The activity of superoxide dismutase (SOD, EC 1.15.1.1) was assessed by the ability to inhibit nitrotetrazolium blue (NBT) photoreduction; the activity of catalase (CAT, EC 1.11.1.6) was determined by the consumption of H_2_O_2_ at 240 nm for 3 minutes, with an extinction coefficient of 36 mM^−1^ cm^−1^. The activity of ascorbate peroxidase (APX, EC 1.11.1.11) was determined by monitoring the oxidation of ascorbate at 290 nm for 3 minutes, with an extinction coefficient of 2.8 mM^−1^ cm^−1^. The activity of guaiacol peroxidase (GPX, EC 1.11.1.7) was determined by the oxidation of guaiacol at 470 nm, with an extinction coefficient of 26.6 mM^−1^ cm^−1^. The activity of glutathione reductase (GR, EC 1.6.4.2) was determined by the oxidation of NADPH at 340 nm for 3 minutes, with an extinction coefficient of 6.2 mM^−1^ cm^−1^. All activities of these enzymes were measured according to the methodology proposed by Garcıa-Limones *et al*.^73^.

The activity of dehydroascorbate reductase (DHAR, EC 1.15.1.1) was determined by the reduction of DHA to ascorbic acid via GSH at 265 nm, with an extinction coefficient of 14 mM^−1^ cm^−1^, according to the methodology proposed by Hossain and Assada^74^.

### Extraction and quantification of L-phenylalanine ammonia lyase (PAL EC 4.3.1.5), tyrosine ammonia lyase (TAL, EC 4.3.1) and phenolic compounds

For enzyme activity, 500-mg aliquots of frozen material were homogenized with 2 mL of 50 mM sodium phosphate buffer, pH 6.5. The material was centrifuged at 4 °C (14000 rpm, 20 minutes), and the supernatant was used as an enzyme extract. The activities were determined by the addition of 50 μL of the enzyme extract at 1 mL of the reaction medium (25 mM Tris-HCl pH 8.8 with 25 mM L-phenylalanine or 25 mM L-tyrosine). The reaction occurred for 1 hour at 37 °C and was quenched by the addition of 60 μL of 6 N HCl. The reaction products, trans-cinnamic (PAL) or p-coumaric (TAL) acid, were determined by reading the absorbance at 290 nm and 310 nm, respectively. The extraction and quantification followed the methodology proposed by Mehta and Bhavnarayana^75^.

For the extraction of phenolic compounds, 500 mg of plant material was macerated in liquid nitrogen, and 2 mL of methanol was added. The material was centrifuged at 14,000 rpm for 30 minutes at a temperature of 20 °C. The supernatant was collected, and the procedure was repeated with the pellet. The samples were read at 720 nm. Quantification was performed by the Folin-Ciocalteau spectrophotometric method through a standard curve of gallic acid, according to the methodology proposed by Singleton *et al*.^76^.

### Extraction and quantification of proline, total soluble sugars, total amino acids and starch

For the extraction of proline, 100 mg of plant material was macerated in liquid nitrogen with 10 mL of 3% sulfosalicylic acid. The solution was placed in tubes and shaken for 60 minutes. After separation of the material, the sample was filtered and analysed according to the methodology proposed by Bates *et al*.^77^.

For soluble sugars, amino acids and starch, 300 mg of plant material was macerated in liquid nitrogen. The extraction was carried out by the addition of the solution consisting of 3 mL methanol, 1,250 mL chloroform and 750 μL water. The samples were left overnight, and the next day, they were centrifuged for 30 minutes at 1300 rpm. The supernatant was collected for analysis of total sugars and amino acids. In the pellet, 1.5 mL of 30% perchloric acid was added.

The material was again left overnight, and the supernatant was collected for starch quantification. The quantification of total sugars and total amino acids was performed according to the methodology described by Gibon *et al*.^78^. Starch was quantified according to the methodology described by Fernie *et al*.^79^.

### Production components

At the end of the experiment (harvesting), the weight of the ear, grain yield and harvest index [dry weight of the grain/(dry weight of the plant + dry weight of the grain)] * 100 were analysed.

### Experimental design and data analysis

The experimental design was randomized block, with 6 treatments: irrigated (IR), water deficit (WD), water deficit with SUC application (WD + SUC), water deficit with the application of MCA (WD + MCA), water deficit with the application of MCA and SUC (WD + MS), and water deficit with the application of chitosan (WD + CHI), with 4 replicates, totalling 24 pots. All analyses were performed at two sampling times (1 and 15 days of water deficit), but the two sampling times were not statistically compared.

For the statistical analysis of the results, the analysis of variance (ANOVA) and Scott-Knott’s comparison test of averages at 0.05% significance (P ≤ 0.05) were run, using Sisvar software 4.3 (Universidade Federal de Lavras, Lavras, Brasil).

## Supplementary information

Video 1
